# Melanoma cells resistant towards MAPK inhibitors exhibit reduced TAp73 expression mediating enhanced sensitivity to platinum-based drugs

**DOI:** 10.1038/s41419-018-0952-8

**Published:** 2018-09-11

**Authors:** Elena Makino, Vanessa Gutmann, Corinna Kosnopfel, Heike Niessner, Andrea Forschner, Claus Garbe, Tobias Sinnberg, Birgit Schittek

**Affiliations:** Division of Dermatooncology, Department of Dermatology, University of Tübingen, Tübingen, Germany

## Abstract

The efficacy of targeted MAPK signalling pathway inhibitors (MAPKi) in metastatic melanoma therapy is limited by the development of resistance mechanisms that results in disease relapse. This situation still requires treatment alternatives for melanoma patients with acquired resistance to targeted therapy. We found that melanoma cells, which developed resistance towards MAPKi show an enhanced susceptibility to platinum-based drugs, such as cisplatin and carboplatin. We found that this enhanced susceptibility inversely correlates with the expression level of the p53 family member TAp73. We show that the lower expression of the TAp73 isoform in MAPKi-resistant melanoma cells enhances accumulation of DNA double-strand breaks upon cisplatin and carboplatin treatment by reducing the efficiency of nucleotide excision repair. These data suggest that a subgroup of melanoma patients with acquired resistance to MAPKi treatment and low TAp73 expression can benefit from chemotherapy with platinum-based drugs as a second-line therapy.

## Introduction

For decades, chemotherapy with dacarbazine (DTIC) was the standard therapy for metastatic melanoma patients despite low tumour remission rates of 5–12%^1,2^. Nowadays, selective kinase inhibitors and immune checkpoint inhibitors are used in the treatment of metastatic melanoma with much higher efficacies. Patients with BRAF-mutated metastatic melanoma treated with inhibitors specific for the mutated BRAF as well as with additional mitogen-activated extracellular signal-regulated kinase (MEK) inhibitors benefit from these therapies^3–5^. However, the development of resistance impedes the long-term efficacy of such targeted therapies. Furthermore, despite the recent success of immunotherapy in the treatment of metastatic melanoma, a subset of patients lacks a positive response^6^. This situation renders chemotherapy still necessary for some metastatic melanoma patients. Currently, chemotherapy can be a treatment option for advanced melanoma patients with secondary resistance to targeted therapy and non-responding to immunotherapy^2^.

Chemotherapeutic drugs are known to activate classical DNA damage sensors, which are related to the p53 signalling pathway^7^ and influence the therapeutic success. In addition to p53, its family member p73 is known to accumulate upon genotoxic drug treatments as well and to influence cellular responses in an isoform-specific manner. Transcripts of the p73 encoding *TP73* gene can be generated from two transcriptional start sites^8^ and undergo further alternative splicing events at the 5′ or 3′ ends, which result in the production of five different N-terminal and at least seven different C-terminal isoforms^8^. The N-terminal TA variants contain the transactivation domain (TAD) and can bind to p53-responsive elements. By this, TAp73 transcriptionally regulates p53 target gene expression as well as the expression of further genes involved in cellular processes, such as cell apoptosis, cell cycle arrest or genome stabilization^9^. There is evidence that the TAp73 isoforms can act either pro- or anti-apoptotic depending on the stress conditions^10^ and promote cancer cell survival in a context-dependent manner^11–14^. Therefore, the precise function of TAp73 and the other p73 isoforms in DNA damage response and tumour survival is still ambiguous. In addition, several studies indicate that the C-terminal composition of the TAp73 isoforms represents an additional determinant for its functional impact^15^. Thus the TAp73β isoform was demonstrated to be responsible for treatment-mediated apoptosis induction in cancer cells including melanoma^15,16^, whereas the TAp73α variant was frequently associated with apoptosis suppression in cancer cells^10,13–15,17^.

Many studies reveal an overexpression of p73 in various cancer types including enhanced expression of the TAp73 isoforms^18^. In metastatic melanoma, it was shown that TAp73 as well as other N-terminal-deleted p73 variants are increasingly expressed during tumour progression^19^. These data implicate that intrinsic p73 expression mediates survival advantages for cancer cells under yet undefined conditions.

In this study, we observed that melanoma cells with acquired resistance to mitogen-activated protein kinase (MAPK) inhibitors (MAPKi) were more susceptible towards carboplatin and cisplatin treatment than the parental sensitive cells. To find a mechanistic explanation for this phenomenon, we analysed the expression of different p53 family members and found that the endogenous level of the TAp73 isoforms were reduced in melanoma cells with acquired resistance to MAPKi. We show that TAp73 influences the DNA damage response to cisplatin and carboplatin via the regulation of nucleotide excision repair (NER). These data suggest that MAPKi-resistant melanoma cells show an enhanced sensitivity towards specific DNA cross-linking agents and that TAp73 activity controls genomic stability and DNA repair in melanoma cells. We propose that the TAp73 expression level might be a possible predictive marker for a subtype of MAPKi-resistant melanoma cells that respond well to cisplatin or carboplatin treatments.

## Materials and methods

### Cell culture

Melanoma cell lines WM3734, 1205 LU, Mel1617 and 451 LU were kindly gifted by M. Herlyn from the Wistar Institute (Philadelphia, USA)^20^. A375, SK-MEL 19 and SK-MEL 28 cell lines were purchased from ATCC. All melanoma cells used were BRAF^V600E^-mutated metastatic melanoma cell lines and exhibit different *TP53* gene mutational status. According to the categories and data previously described and available at *IARC TP53* data base^21^, A375, WM3734, 1205Lu and Mel1617 are *TP53* wild-type cell lines, *TP53* mutation of the SK-MEL 28 (*TP53* L145R) and 451Lu (*TP53* Y220C) cell line leads to the expression of a non-functional p53 protein and the *TP53* mutation of the SK-MEL 19 cell line (*TP53* N131K) still enables partially functional p53 protein expression. Melanoma cell lines were cultured as described previously^22^. MAPKi-resistant melanoma cell lines were generated by treatment with continuously increasing concentration of the BRAF inhibitor (BRAFi) vemurafenib (up to 2 µM) or additionally with the MEK inhibitor (MEKi) trametinib (up to 50 nM) for several months.

### Viability assay

4-Methylumbelliferyl heptanoate (MUH) assays were performed as previously described^22^. The cells were treated with cisplatin (Hexal, up to 20 µM), carboplatin (Medac, up to 250 µM), vemurafenib (LC Laboratories, up to 20 µM), trametinib (Selleck Chemicals, up to 250 nM), 5-fluorouracil (R&D Systems, up to 25 µM), paclitaxel (Merck, up to 500 nM) or temozolomide (TMZ; Merck, up to 50 µM) for 72 h.

### Cell cycle analysis

Cell cycle analysis was performed as described previously^22^. The cells were treated with cisplatin (2.5 or 5 µM) or carboplatin (25 or 50 µM) for 72 h.

### Small interfering RNA (siRNA) transfection

The siRNAs were transfected using Lipofectamine RNAiMAX Reagent (Thermo Fisher Scientific) according to the manufacturer’s protocol. In all, 2.5 × 10^5^ cells were seeded into each cavity of a 6-well plate 24 h before the transfection. siRNA (25 nM) were transfected for 24 h in 1.5 ml Opti-MEM Medium. One ml of RPMI 1640 Medium (Gibco Life Technologies) supplemented with 10% foetal calf serum (Biochrom/Merck Millipore) was added 6 h after transfection start. Subsequently, the cells were seeded for the respective experiments (MUH viability assay, cell cycle analysis, immunofluorescence staining and western blot analysis) or cells were collected for reverse transcriptase–quantitative PCR (RT-qPCR) analysis. All siRNAs were synthesized by biomers.net. Following siRNA sequences were used: si1 TAp73 sense: 5′-GCACCACGUUUGAGCACCU-dTdT-3′, antisense: 5′-AGGUGCUCAAACGUGGUGC-dTdT-3′; and si2 TAp73 sense: 5′-GGAACCAGACAGCACCUACUU-dTdT-3′, antisense: 5′-AAGUAGGUGCUGUCUGGUUCC-dTdT-3′.

### Plasmid transfection

The cells were transfected with HA-TAp73α encoding plasmid (Addgene) and the pcDNA3 control plasmid (Addgene) using Lipofectamine 3000 (Thermo Fisher Scientific) according to the manufacturer’s protocol. In all, 2.5 × 10^5^ cells per well were seeded 24 h prior to the transfection and the transfection was performed for 6 h. After further 24 h, the cells were collected for RT-qPCR analysis or again seeded for viability assays. The samples for the immunoblot analysis were collected 48 h after the transfection.

### Anchorage-independent growth assay

In all, 1.5 × 10^3^ cells were embedded in 0.5% agarose-medium mixture in each cavity of a 12-well plate. The cells in the soft agar were treated with cisplatin (5 µM) containing growth culture medium. The control cells were grown in normal growth culture medium. The cells were incubated for 12 days at 37 °C and the respective medium was changed every 3–4 days. The cell colonies were subsequently stained with 0.05% crystal violet solution in 20% phosphate-buffered saline (PBS) and 80% methanol overnight. Melanoma cell colonies in 5 microscope fields of vision (×200) were counted for each well and each experiment was performed in triplicates.

### Clonogenic assay

For the analysis of melanoma cell growth, 8 × 10^3^ (for 5-day treatment) or 1.5 × 10^4^ (for 3-day treatment) cells were seeded in each cavity of 24-well plates and the treatment was started 24 h later. The cells were treated with different concentrations of cisplatin (2.5, 5, 10 µM for 3 days or with 0.5, 1 µM for 5 days). Formalin 4% was used for the fixation and the cells were subsequently stained with 3% crystal violet solution (Sigma-Aldrich) in 50% methanol and 50% PBS.

### Immunofluorescence staining

Immunofluorescence staining was performed as described previously^22^. In all, 2.5 × 10^4^ cells were seeded in 4-well chamber slides (BD Falcon). The cells were treated with cisplatin (5 µM) for 24 h and stained for pH2AX (anti-pH2AX antibody, Abcam, 1:100). The pH2AX foci of 50–100 cells were counted for each sample.

### Immunoblot analysis

Whole-cell lysates of melanoma cells and nuclear-enriched cell lysates were prepared and immunoblot analysis was performed as described previously^22^. Fifteen μg protein of whole-cell lysate or 5 μg of nuclear-enriched lysates were used for the sodium dodecyl sulphate–polyacrylamide gel electrophoresis. The following primary antibodies were used: anti-Lamin-B (C-20, Santa Cruz Biotechnology, 1:250), anti-p53 (DO-I, Santa Cruz Technology), anti-Actin (D6A8, CST, 1:1000), anti-p73 (GC-15, BD Biosciences, 1:250; H-79, Santa Cruz, 1:250; 5B429, Novus Biologicals, 1:500), anti- pH2AX (JBW301, Abcam, 1:500), anti-Mdm2 (Abcam, 1: 100), anti-p21 (CST, 1:2000), and anti-HA (Santa Cruz, 1:250).

### RT-qPCR analysis

RNA was isolated using the Nucleospin RNA Kit (Macherey-Nagel). cDNA was generated using Maxima reverse transcriptase (Thermo Fisher Scientific) according to the manufacturer’s protocol. RT-qPCR was performed using KAPA SYBR Fast qPCR Master Mix (Peqlab) and the Lightcycler 480 (Roche). The primer sequences are listed in Supplementary Table 1.

### Flow cytometric analysis for cisplatin adducts

Melanoma cell lines were treated with cisplatin (10 μM) for 6 h and incubated in fresh medium for additional 3 or 6 h. The collected samples were fixed in 1 ml 70% Ethanol at 4 °C for at least 1 h and the cells were permeabilized with 0.5% Triton-X for 30 min. The samples were stained using anti-cisplatin-modified DNA antibody (CP9/19, Abcam, 1:100) or anti-rat IgG control (Thermo Fisher Scientific, 1:100) diluted in PBS overnight at 4 °C. The samples were additionally incubated in fluorescein isothiocyanate (FITC)-conjugated donkey anti-rat IgG H&L secondary antibody (Abcam, 1:100) for 2 h at room temperature and subsequently in 100 μg/ml RNAse A (Applichem) and 50 μg/ml propidium iodide (PI; Sigma) for 15 min. The analysis of the cell cycle phases and the FITC-labelled cisplatin adducts were performed with LSRII FACS (BD) using the FACSDiva software (BD). The cells in the G1 phase were determined by the PI staining data and the mean fluorescence intensity of these cells were calculated using the FlowJo software (FlowJo LLC).

### PCR analysis

The levels of six different C-terminal p73 isoform transcripts were analysed according to a published method^23^. Gel electrophoresis was performed using a 1.5% agarose gel stained with GelRed Nucleic Acid Gel stain (ChemoMetec). The gels were run for 2 h at 150 V.

### Patient data

Cutaneous metastatic melanoma patients who received a targeted therapy with MAPKi and subsequently a carboplatin/paclitaxel therapy between June 2013 and July 2015 at our institution with available follow-up data were included in the analysis. This retrospective analysis was approved by the Ethics committee Tübingen, Germany (approval number 800/2016B02). The approval for this study was gained retrospectively. The chemotherapeutic treatment consisted of intravenous paclitaxel 225 mg/m^2^ and intravenous carboplatin at area under curve 6 (AUC 6), which was applied on day 1 of a 21-day cycle. After the fourth cycle, the treatment dose was reduced to carboplatin AUC 5 and paclitaxel 175 mg/m^2^. Patients in poor general condition or with insufficient myelofunction received a reduced dose. Tumour response was evaluated using the Response Evaluation Criteria in Solid Tumours ^24,25^.

### Database analysis

The relative *TP73* gene expression was analysed in a published mRNA expression profiling data set^26^ of melanoma patient samples. The fold change of log2-transformed signal intensities of the MAPKi-treated melanoma patient samples are shown in relation to the appropriate signal intensities of the tumour samples collected from the corresponding patients before therapy start.

### Statistical analysis

Microsoft Excel and Graphpad Prism 6.0 were used for the statistical analyses. The mean values with their standard deviation (SD) are shown if not indicated differently. The data points were compared using multiple *t* tests with Holm–Šídák correction if not indicated differently. *P* values <0.05 were considered as statistically significant and are indicated in the graphs by asterisk/s (ns: *p* > 0.05, *p < 0.05, **p < 0.01, ***p < 0.001). Dose–response curves were fitted using Graphpad Prism 6.0 as described previously^22^.

## Results

### MAPKi-resistant melanoma cells are susceptible to platinum-based drugs

**Fig. 1:**
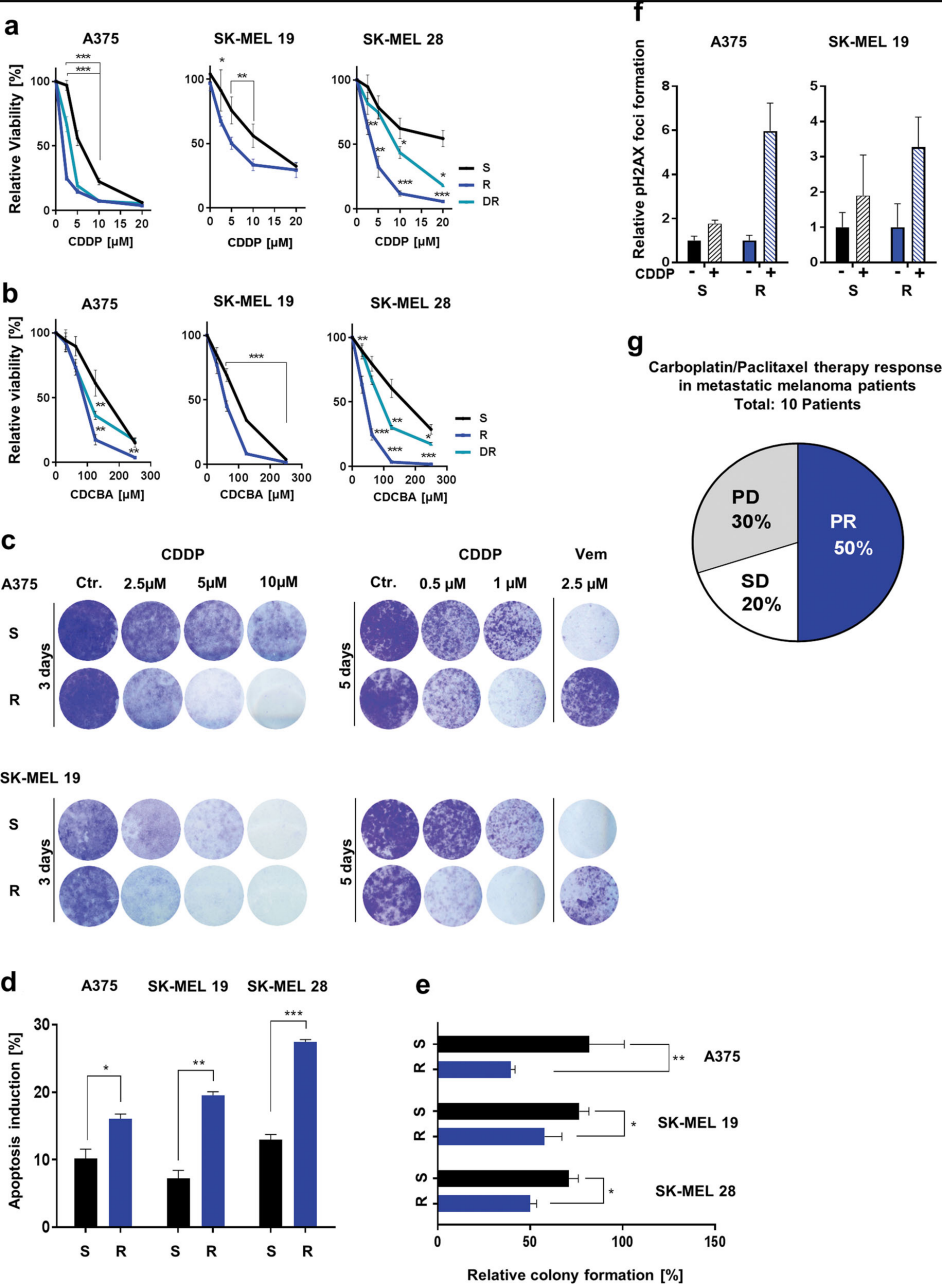
MAPKi-resistant melanoma cells are susceptible to cisplatin and carboplatin treatment. **a**, **b** Viability analysis after the treatment of treatment-naive (S), BRAFi-resistant (R) and BRAFi and MEKi double-resistant (DR) melanoma cells with cisplatin (CDDP, up to 20 µM) (**a**) or carboplatin (CDCBA, up to 250 µM) for 72 h (**b**). The viability signals were normalized to untreated cell signals. (Mean values ± SD, *n* = 3) **c** Clonogenic assay of S and R melanoma cells after cisplatin (CDDP) treatment for 3 or 5 days (crystal violet staining). **d** Percentage of apoptosis induction 3 days after cisplatin (5 µM) treatment of S and R melanoma cells. (Mean values ± SD, *n* = 3). **e** Relative number of cell colonies formed in soft agar 12 days after cisplatin treatment (5 µM) by S and R melanoma cells. The colony numbers were normalized to the number of colonies formed by corresponding untreated cells. (Mean values ± SD, *n* = 3). **f** pH2AX foci number per cell counted in samples of S and R melanoma cells after cisplatin treatment (CDDP, 10 µM) for 24 h. pH2AX foci number per cell was normalized to the number of foci per untreated control cell (Ctr.). (Mean values ± SD; *n* = 3). **g** Patient response to carboplatin/paclitaxel therapy denoted as progressive disease (PD), stable disease (SD) or partial response (PR) was analysed using data of ten melanoma patients. All patients received a previous targeted therapy shown in Suppl. Figure 2a

We analysed the chemosensitivity of melanoma cells with acquired resistance to BRAF inhibitor (vemurafenib) (R) or combined BRAF and MEK inhibitor (vemurafenib and trametinib) (DR) treatment and of their sensitive (S) counterparts. Surprisingly, we observed an enhanced susceptibility of all R and DR cells towards cisplatin and carboplatin (Fig. 1a, b, Suppl. Figure 1A) in comparison to the parental S cells but not towards treatment with other chemotherapeutic drugs, such as 5-fluorouracil, temozolomide and paclitaxel (Suppl. Figure 1B). The augmented cisplatin sensitivity of the R cells was further confirmed in a clonogenic assay (Fig. 1c). In addition, cell cycle analyses indicated that apoptosis induction was enhanced in R cells after cisplatin treatment as well (Fig. 1d). We further used three-dimensional culture assays to analyse the effect of chronic cisplatin or carboplatin treatment on melanoma cells in a more physiological system and found that anchorage-independent growth was significantly reduced in the BRAFi-resistant melanoma cells 12 days after cisplatin treatment (Fig. 1e). We additionally analysed the DNA double-strand break accumulation 24 h after cisplatin treatment via pH2AX staining. As shown in Fig. 1f, BRAFi-resistant cells accumulated a higher number of pH2AX foci than the sensitive cells.

In a clinical setting, these observations would imply that treatment of melanoma patients developing a resistance to MAPKi therapy have a better response to platinum-based chemotherapeutic agents. Clinical data support this assumption since a positive response (stable disease or partial regression) to carboplatin/paclitaxel therapy was observed in 7 of the 10 patients who suffered from a relapse after MAPKi treatment (Fig. 1g, Suppl. Figure 2A). In comparison to data of studies conducted before the approval of targeted melanoma therapy, these data denote a relatively higher responder proportion in the MAPKi-resistant melanoma patients towards carboplatin/paclitaxel therapy^27^. In three of these patients, combined chemotherapy including carboplatin resulted in a clear positive response, which was reflected by decreased levels of the tumour marker S-100 or lactate dehydrogenase (LDH) (Suppl. Figure 2B). These data indicate that MAPKi-resistant melanoma cells in vitro and in patients are sensitive to platinum-based chemotherapeutic agents, presumably due to an increased accumulation of DNA double-strand breaks.

### MAPKi-resistant melanoma cells show a reduced expression of the TAp73 isoforms

**Fig. 2:**
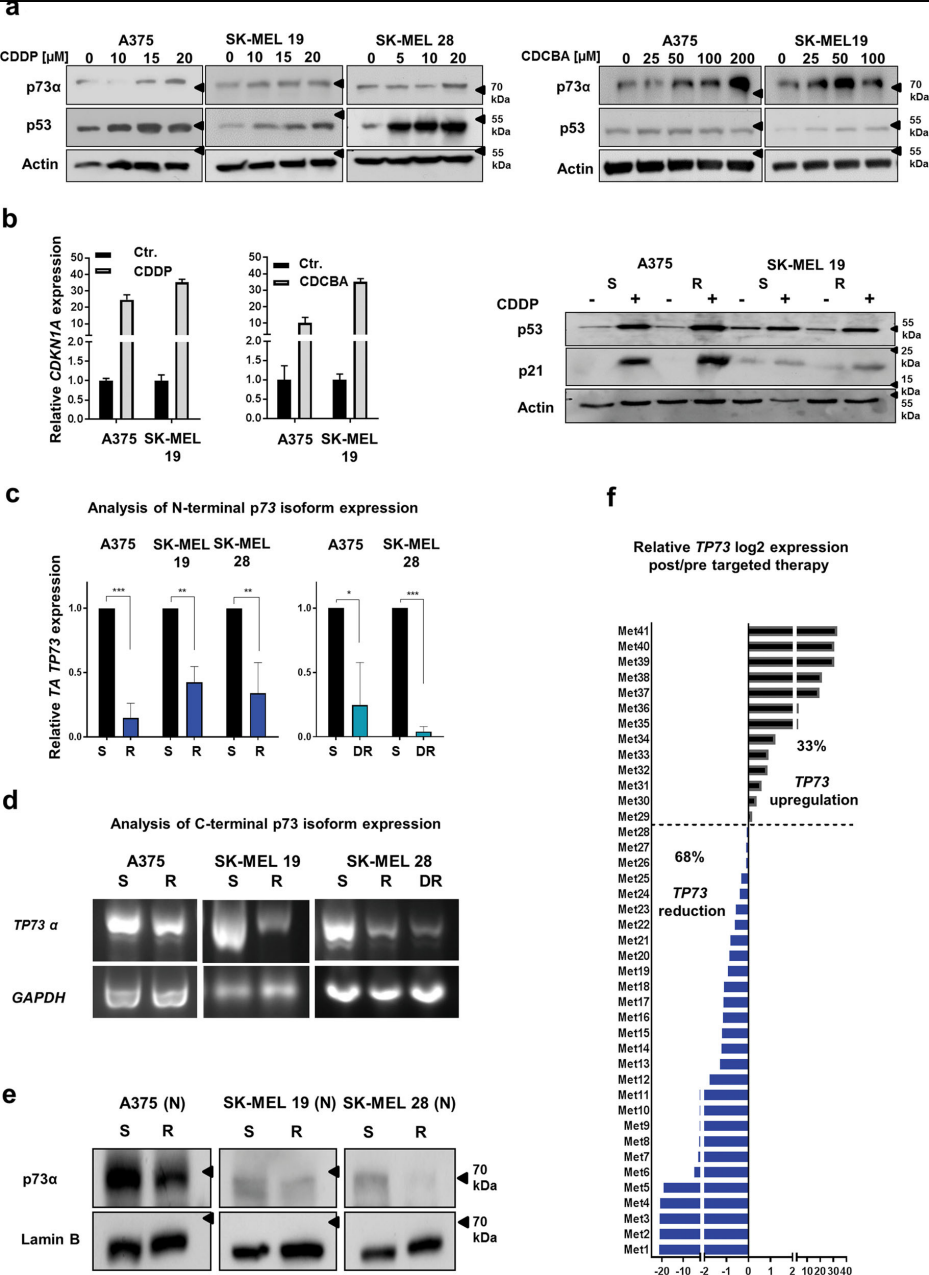
TAp73α levels are reduced in MAPKi-resistant cells. **a** Immunoblot analysis for p73α and p53 in different S melanoma cells after treatment with the indicated concentrations of cisplatin (CDDP) or carboplatin (CDCBA) for 24 h. Actin served as a loading control. **b** Left graphs: p21 mRNA expression (*CDKN1A)* after cisplatin (CDDP) or carboplatin (CDCBA) treatment in S melanoma cells normalized to Actin expression and to the corresponding untreated control cells (Ctr.) set as 1. Exemplary data of one experiment performed in triplicates are shown. Right graphs: Immunoblot analysis of p53 and p21 protein expression was performed in S and R melanoma cell lines with or without cisplatin treatment (5 µM) for 24 h. Actin served as a loading control. **c** TAp73 mRNA expression (*TA TP73*) in S, R and DR melanoma cell lines normalized to Actin expression and to the corresponding S cells set as 1 (Mean values ± SD; *n* = 3). **d** Detection of C-terminal *p73α* isoform transcript levels in S, R and DR melanoma cells. GAPDH expression was used as a control (PCR and subsequent agarose gel electrophoresis). **e** Immunoblot analysis of p73α protein in nuclear enriched lysate fractions (N) of S and R melanoma cells. Lamin B served as a loading control. **f** Published data set of mRNA sequencing performed by Hugo et al.^26^ was used for the analysis. *TP73* expression (log2 signals) in metastatic melanoma samples under MAPKi treatment was normalized to the *TP73* expression in the metastases collected before treatment start from the same patient, respectively

We found that cisplatin and carboplatin treatment results in an increased expression of the p53 protein family members p53 and p73α and their target gene *CDKN1A* (p21) in melanoma cells (Fig. 2a, b). The p63 protein was not present on a detectable level in these cells with or without cisplatin treatments (data not shown). Therefore, we focussed on the influence of p53 and p73 on chemosensitivity in our further analyses.

First, we asked whether the enhanced platinum-based drug sensitivity of MAPKi-resistant cells is based on an alteration of the p53 signalling pathway activity in R cells. Surprisingly, the protein level of p53 and its target p21 did not differ between MAPKi-sensitive and -resistant melanoma cell lines neither on a basal level nor after cisplatin treatment (Fig. 2b, Suppl. Figure 3A). We went on to analyse the basal transcript levels of p73 N-terminal isoforms. To this end, RT-qPCR analysis was performed using a specific set of primers recognizing the different 5′-ends of the *TP73* transcripts (Suppl. Table 1; Suppl. Figure 3B). We found that all examined MAPKi-resistant melanoma cell lines exhibit a significantly lower basal expression of the TAp73 isoforms (*TA TP73*) in comparison to the corresponding MAPKi-sensitive cells (Fig. 2c). In contrast, the expression of other N-terminal p73 isoforms did not consistently differ between the S and R cell line pairs (Suppl. Figure 3C). We additionally found that the TAp73 isoform class is one of the most abundantly expressed N-terminal isoform class followed by the ΔNEx2/3p73 variants (Suppl. Figure 3D). Furthermore, we analysed the *TP73* gene transcripts regarding the level of C-terminal isoform classes (α–ζ) using a 3′-end-specific primer pair (Suppl. Figure 3B). We found that the expression of the p73α isoform class is remarkably reduced in MAPKi-resistant cells (Fig. 2d). On protein level, we confirmed a prominent expression of the TAp73α isoform among different C-terminal isoform classes using two different antibodies (Suppl. Figure 3E). In addition, we show that the expression of p73α on nuclear protein level is reduced in R cells in comparison to the corresponding S cells (Fig. 2e).

We additionally used the mRNA sequencing data published by Hugo et al. in which tumour samples from melanoma patient both before and after the acquisition of MAPKi resistance were analysed^26^. The analysis revealed that nearly two thirds of metastatic melanoma samples (29 of 43) collected after MAPKi-resistance development had a reduced expression of p73 mRNA relative to the level in the corresponding tumour samples collected before the therapy start from the same patient (Fig. 2f). These data strongly suggest that MAPKi-resistance development in a subset of melanomas correlates with a reduction of p73 mRNA expression, which is most likely mediated by the lower expression of the intrinsically highly expressed N-terminal TAp73 and C-terminal p73α isoform.

### TAp73 levels determine the susceptibility to cisplatin treatment

**Fig. 3:**
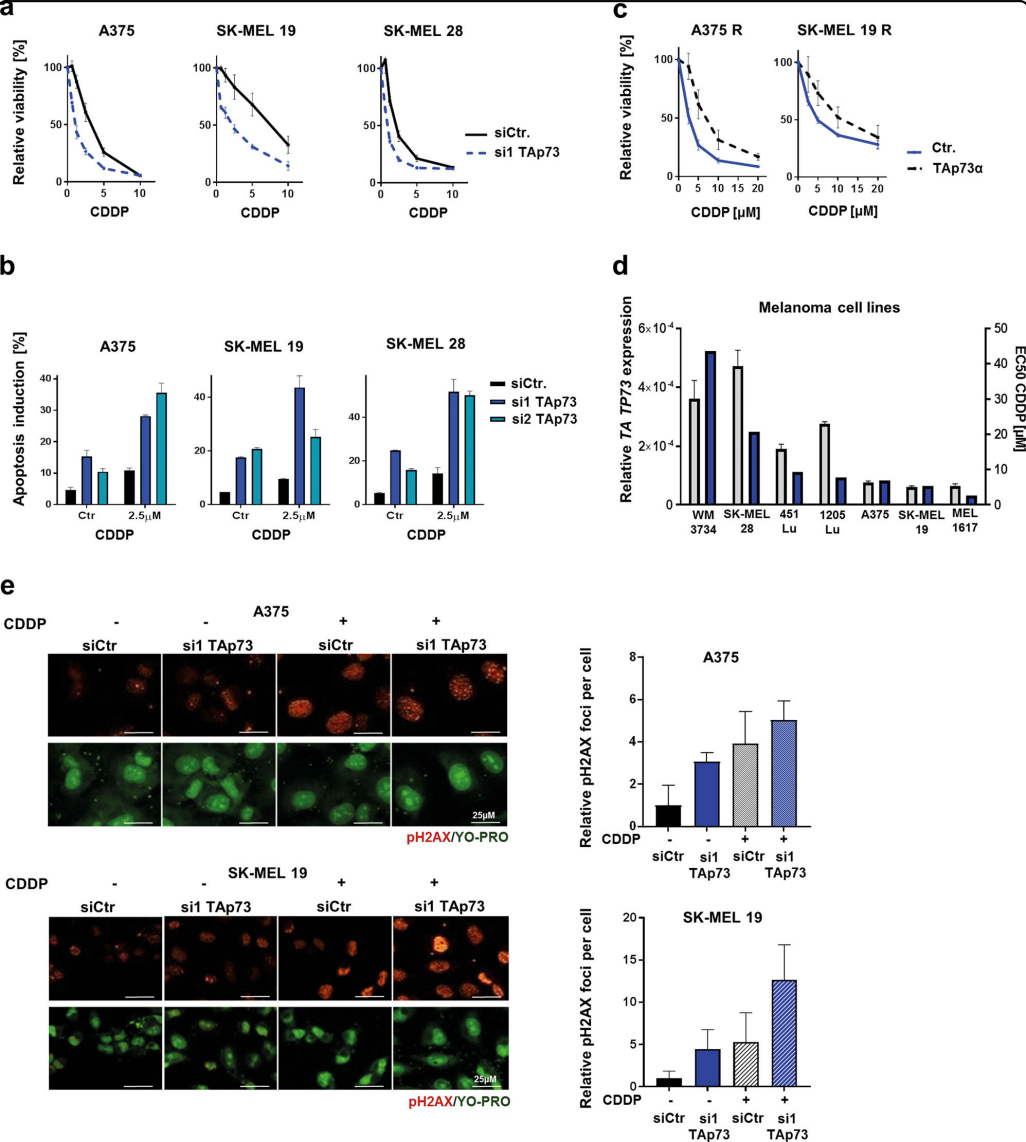
TAp73 downregulation sensitizes melanoma cells towards cisplatin and carboplatin treatment. **a** Viability analysis was performed in S melanoma cell lines after the transfection of siRNA against TAp73 (si1 TAp73) or non-silencing control siRNA (siCtr.) and the additional treatment with up to 20 µM cisplatin (CDDP) for 72 h. The assay signals were normalized to the appropriate untreated cell signals. Exemplary data of one experiment with five replicates are shown. (Mean values ± SD). **b** Apoptotic cell fraction (in %) was analysed after the treatment of siRNA-transfected melanoma cell lines (si1TAp73, si2TAp73, siCtr.) with 2.5 µM cisplatin (CDDP) for 72 h via cell cycle analysis. Transfected and untreated cell samples were used for the detection of basal apoptotic cell fractions (Ctr.). Exemplary data of one experiment with triplicates are shown. (Mean values ± SD). **c** Viability analysis was performed on R melanoma cells transfected with HA-TAp73α plasmid (TAp73α) or pcDNA3 control-plasmid (Ctr.), with or without treatment with cisplatin (CDDP, up to 20 µM) for 72 h. Assay signals were normalized to untreated cell signals. Exemplary data of one experiment with five replicates are shown. (Mean values ± SD). **d** Comparison of cisplatin EC50 values (blue bar) and *TA TP73* expression (grey bar) in metastatic melanoma cell lines. TAp73 mRNA expression (*TA TP73*) was normalized to Actin expression (Mean values ± SD; *n* = 3). EC50 values were analysed using the data of the appropriate viability assays (*n* = 3) after cisplatin treatment (up to 20 µM) for 72 h. **e** Representative pictures of pH2AX (in red) and YO-PRO (in green) staining in S melanoma cells transfected with siRNA against TAp73 (si1 TAp73) or control siRNA (siCtr.) with or without additional cisplatin treatment (CDDP, 24 h, 5 µM). The white bar represents 25 µm. Right graph: pH2AX density (foci/cell) after cisplatin treatment was normalized to the basal pH2AX density in the appropriate cells without treatment set as 1 (Mean values ± SD, *n* = 3)

To analyse whether the observed reduction of TAp73 levels in MAPKi-resistant melanoma cells is responsible for the enhanced susceptibility towards cisplatin and carboplatin treatment, we downregulated TAp73 expression in treatment-naive melanoma cells via transfection of two specific siRNAs (Suppl. Figure 4A). We observed that TAp73 knockdown does not influence the sensitivity towards the BRAFi vemurafenib (Suppl. Figure 4B) but significantly sensitizes melanoma cells towards cisplatin and carboplatin treatment (Fig. 3a, Suppl. Figure 4C) and enhances apoptosis induction (Fig. 3b, Suppl. Figure 4D). Of note, the enhanced sensitivity of the melanoma cells towards cisplatin and carboplatin was independent of the *TP53* mutation status of the different cell lines (see Materials and methods). We further induced an overexpression of HA-tagged TAp73α in the MAPKi-resistant melanoma cell lines with low basal TAp73 expression (Suppl. Figure 4E), which resulted in a clear enhancement of resistance towards cisplatin treatment (Fig. 3c) but did not affect the sensitivity towards the BRAFi vemurafenib (Suppl. Figure 4B). Furthermore, we analysed the effective concentration of cisplatin able to reduce 50% of cell viability (EC50 levels) in different human BRAF^V600E^ metastatic melanoma cell lines and found a correlation to the basal level of TAp73 mRNA expression (Fig. 3d). All these data indicate that TAp73 mediates resistance towards cisplatin treatment in melanoma cells.

Next, we analysed the influence of TAp73 levels on cisplatin-mediated DNA double-strand break accumulation via detection of H2AX phosphorylation^28^. We observed an enhanced number of pH2AX foci per cell in cisplatin-treated TAp73 knockdown cells in comparison to the cisplatin-treated control siRNA-transfected cells (Fig. 3e). These data strongly indicate that TAp73 has a protective function in melanoma cells reducing the cytotoxicity of platinum-based drug treatments by the limitation of DNA double-strand break accumulation.

### DNA damage repair is impaired in MAPKi-resistant melanoma cells in a TAp73-dependent manner

**Fig. 4:**
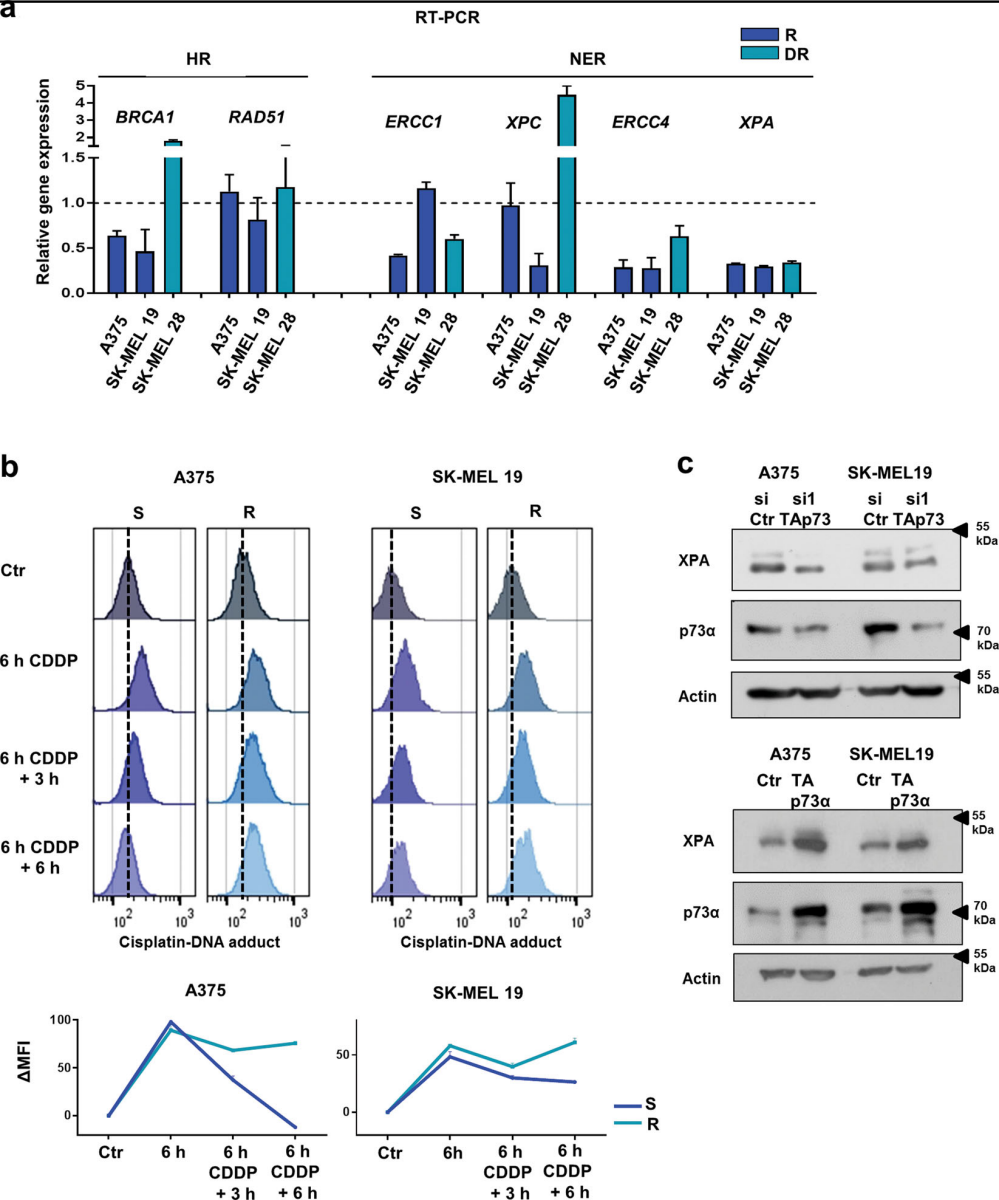
NER is impaired in MAPKi-resistant cells and is influenced by the TAp73 level. **a** Expression of homologous recombination (HR) or nucleotide excision repair (NER)-related mRNA in R and DR melanoma cell lines compared to gene expression in S cells set as 1. Gene expression was normalized to the corresponding *Actin* expression (Mean values ± SD; *n* = 3). **b** Cisplatin adduct levels of G1 phase cells were analysed after the treatment of S and R melanoma cells with cisplatin (CDDP, 10 μM) for 6 h as well as after additional incubation of these cells in drug-free medium for 3 h and 6 h (6 h CDDP +3 h or 6 h CDDP + 6 h). Control cells (Ctr) were untreated. Shown are representative histograms (upper graphs). Mean fluorescence intensity (MFI) indicates the mean cisplatin adduct level per cell measured in triplicates (lower graphs). Exemplary data of one experiment is shown. **c** Immunoblot analysis for XPA, p73α and Actin was performed using lysates of S melanoma cells 48 h after the transfection with siRNA against TAp73 (si1 TAp73) or control siRNA (siCtr) (upper blots) or using lysates of R melanoma cells, which were collected 48 h after the transfection of HA-TAp73α-expressing plasmid (TAp73α) or pcDNA3 control-plasmid (Ctr) (lower blots)

Since we saw an enhanced accumulation of DNA double-strand breaks upon the cisplatin treatment in MAPKi-resistant cells, we asked whether there are differences in the DNA repair capacity between the MAPKi-resistant and -sensitive cells. Cisplatin- and carboplatin-mediated DNA cross-links are primarily removed by NER in the G1 phase and secondarily by homologous recombination (HR) upon DNA double-strand break induction^29^. Therefore, we first determined the basal transcript levels of four critical genes involved in NER (*ERCC1*, *ERCC4*, *XPA*, *XPC*) and of two genes known to be involved in HR repair (*BRCA1*, *RAD51*) in the MAPKi-sensitive and -resistant cells. We did not find a consistent difference in the basal expression of HR-related gene transcripts; however, the basal expression of *XPA* and *ERCC4* involved in NER was consistently lower in MAPKi-resistant cells compared to the MAPKi-sensitive cells (Fig. 4a). To evaluate the efficacy of NER in MAPKi-sensitive and -resistant melanoma cells, we analysed the kinetics of cisplatin-mediated DNA adduct removal upon cisplatin treatment by flow cytometric detection of DNA adducts over time. We could show that the DNA adducts in the G1 phase cells generated upon 6 h of cisplatin treatment were completely removed in the MAPKi-sensitive cells 6 h later. In contrast, repair of the G1 phase cell DNA adducts in MAPKi-resistant melanoma cells was insufficient as the DNA adduct level was retained 6 h later (Fig. 4b). These data show that the enhanced accumulation of DNA double-strand breaks upon platinum-based drug treatment of MAPKi-resistant melanoma cells is mediated by a reduced NER capacity in these cells.

To finally analyse whether TAp73 is critically involved in DNA damage repair after genotoxic treatment, we downregulated the TAp73 expression in sensitive melanoma cells and analysed the mRNA expression of genes involved in HR and NER (Suppl. Figure 4F). We found that especially the expression of *XPA* and *ERCC4*—genes associated with NER and downregulated in MAPKi-resistant cells—are affected by TAp73 downregulation. In addition, we could show that protein levels of Xeroderma Pigmentosum group A (XPA) were downregulated by siTAp73 expression and upregulated by TAp73α overexpression in these cells (Fig. 4c). These data indicate that TAp73 influences NER in melanoma cells by increasing the level of NER-related proteins. In MAPKi-resistant melanoma cells, TAp73 expression is lower resulting in a reduced NER efficacy and an enhanced sensitivity towards platinum-based cytostatic agents.

## Discussion

**Fig. 5:**
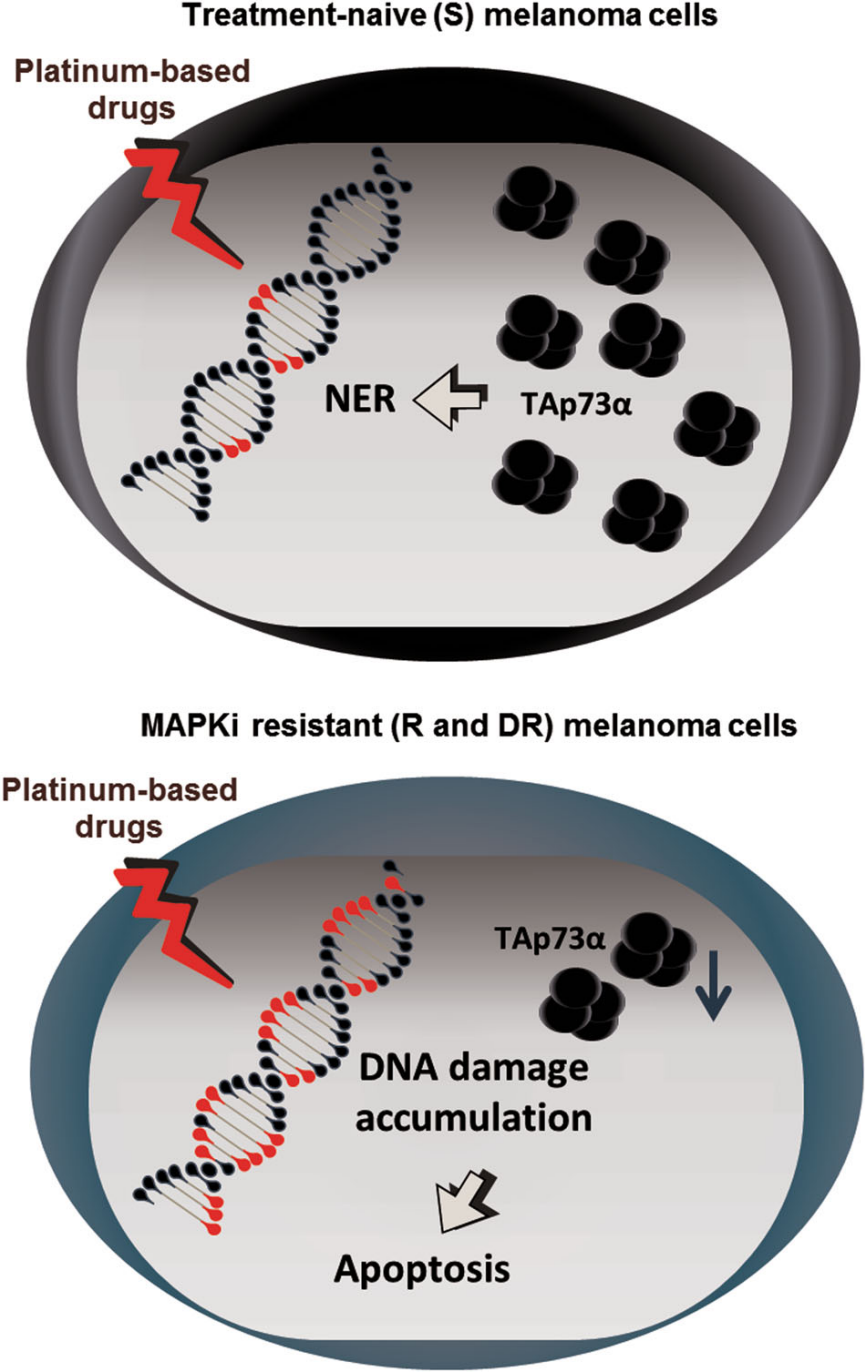
The mechanism of cisplatin sensitization upon MAPKi-resistance acquisition in metastatic melanoma cells. The level of TAp73α negatively influences the sensitivity towards cisplatin and carboplatin treatment by regulation of NER in melanoma cells. A subset of MAPKi-resistant melanoma cells have a reduced level of TAp73α and are thereby sensitive to apoptosis induction mediated by platinum-based drug treatments

In our study, we showed that MAPKi-resistant melanoma cells exhibit an enhanced susceptibility towards platinum-based cytostatic agents. We identified that these cells have a lower basal level of the TAp73 mRNA, which results in an enhanced DNA damage accumulation and reduced NER (Fig. 5). These data suggest that melanoma cells that develop an acquired resistance towards targeted therapies are specifically vulnerable to DNA-damaging drugs, such as cisplatin and carboplatin.

The development of molecularly targeted therapies such as the BRAFi vemurafenib or dabrafenib^3,4^ and especially the combined BRAF and MEK inhibition improved the prognosis for BRAF-mutated metastatic melanoma patients^5^. Nevertheless, the majority of patients show progressive disease even after combined inhibitor treatment due to resistance development. Mechanisms of resistance to BRAFi or to combined BRAFi and MEKi treatment have been extensively investigated^5^. Currently, several studies are ongoing that aim to overcome the resistance towards targeted MAPKi therapy in melanoma^30,31^. However, these strategies only achieve the delay of resistance development but cannot completely avoid a relapse so far. Therefore, the treatment with chemotherapeutic agents is to be considered as an option for patients with acquired MAPKi resistance non-responding to approved immunotherapies. Here we show an enhanced susceptibility to platinum-based drugs in a subset of MAPKi-resistant melanoma cell lines with reduced TAp73 mRNA expression. We additionally observed that polychemotherapy with carboplatin and paclitaxel of some patients developing resistance towards MAPKi therapy strongly reduced the LDH and S-100 levels, which reflects a positive response to chemotherapy. Former clinical studies of melanoma patient treatments with combined application of carboplatin and paclitaxel in a common 3-week regimen showed a partial regression rate of 5–26%^27^. In contrast, the present retrospective data from our clinic demonstrates that at least 5 out of the 10 targeted therapy-treated melanoma patients (50%) could benefit from this polychemotherapy by partial remission. Although the patient number is low, these data underline the potential of carboplatin-containing polychemotherapy as a clinical treatment strategy for MAPKi-resistant melanomas, which has to be followed up in clinical studies in future.

We further explored the underlying mechanism explaining the correlation between the TAp73 downregulation and the sensitivity to platinum-based drugs. Similar to p53, p73 is stabilized upon DNA damage induction and is able to mediate a pro-apoptotic response^32^. The *TP73* transcripts undergo alternative splicing events, which result in transcription and translation of a broad range of C- and N-terminal isoforms^8^. Within this work, we could show that TAp73 belongs to the most abundant N-terminal isoform transcripts and p73α variant forms the predominant C-terminal isoform in melanoma cell lines. The upregulated expression of the TAp73 isoforms as known for melanoma cells^19^ was already shown in several further cancer types such as neuroblastoma, hepatocellular carcinoma or ovarian carcinomas^15,33^ hinting at their oncogenic properties.

In melanoma cells, the pro-apoptotic impact of an ectopic TAp73β expression in response to cytostatic treatments has been extensively demonstrated^15,16^. However, our data show that the predominant p73α isoform inversely acts in a melanoma cell-protective manner upon cisplatin or carboplatin treatment. In line with our observations, several studies confirm a chemo-protective function of p73α in different cancer cell types^10,13–15,17^. These results support the importance of C- and N-terminal isoform discrimination upon the functional analysis of p73.

One known cancer cell-protective activity of TAp73 is mediated by its inhibitory influence on p53 activity^10^. This would implicate that the observed chemo-sensitization upon TAp73 downregulation could be mediated via a reduction of the p53 activity blockade. Similarly, a p53 inhibitory influence is commonly reported for the ΔNp73 variants also in melanoma cells^34^ as well as for the p73α isoform in another cancer cell setting^17^. In addition, different proteins can interact with the α isoform-specific SAM domain or the extreme C-terminus of p73α. Both domains have the capacity to independently inhibit the p53 transcriptional activity by influencing the target promoter binding^13,35^. However, since we found the chemo-sensitizing effect of TAp73 knockdown in melanoma cells with different *TP53* mutational status and activity, it is to be assumed that TAp73 acts independently of its crosstalk with p53-mediated signalling in the protection of melanoma cells to cisplatin or carboplatin treatment. We could additionally show that the expression and activity of p53 did not differ upon cisplatin treatment between MAPKi-sensitive and -resistant cells supporting that p53 is not implicated in the sensitization of MAPKi-resistant cells to platinum-based drugs.

It is known that some genes specifically regulated by p73 or its family member p63 are involved in DNA repair mechanisms^36^. Moreover, it was shown that especially the TAp73 isoform expression can be critical for the maintenance of genomic stability in a cell line-specific manner^37^. These reports are in accord with the DNA damage induction upon TAp73 knockdown and the enhanced susceptibility of the TAp73-low MAPKi-resistant melanoma cells to cisplatin or carboplatin treatment. Cisplatin mediates its genotoxicity by the covalent binding to purine bases of the DNA, which mainly generates inter- and intra-strand adducts^38^. These DNA adducts can inhibit replication and induce cellular apoptosis in the absence of adequate repair machinery activity. NER is the repair machinery primarily responsible for the removal of cisplatin- or carboplatin-mediated intra-strand adducts^29^. Secondarily, DNA double-strand breaks can be induced upon inter-strand adducts removal or as a consequence of unrepaired DNA damage. These double-strand breaks require the HR mechanism^29^ for adequate error-free DNA repair. The detected sensitization to anticancer drug treatment going along with the BRAFi resistance was seen for the treatment with platinum-based drugs, however, not for other agents including paclitaxel and 5-fluorouracil. Significant differences in temozolomide susceptibility were only seen in one R and S cell line pair. Among these agents, platinum-based drugs especially mediate the cytotoxicity via DNA adducts formation that primarily require NER for the removal. The NER alteration in the BRAFi-resistant cells substantiates this selective sensitivity profile.

In line with this, we found a direct correlation between TAp73 levels and the expression of the NER-associated protein XPA. Indeed, augmented expression and activity of NER repair-related proteins has been commonly reported to mediate cisplatin resistance^38,39^. Several ongoing approaches aim at targeting the rate-limiting DNA damage incision step of the NER in cancer cells, which is mainly accomplished by the proteins XPG, XPF, XPA, RPA and ERCC1^40^. The NER-associated XPA protein was already recently shown to mediate chemo-resistance in melanoma^41^ in a non-DNA repair-related context. In addition, the NER-associated gene *ERCC1* has been reported to be an attractive target in melanoma in order to overcome cisplatin-resistance development^42,43^. Further approaches are needed to analyse whether TAp73 directly or indirectly regulates the expression of NER components on transcript or protein level, especially in the context of XPA expression and which genome-stabilizing mechanisms rely on TAp73 expression in melanoma cells.

The remaining question is the mechanism underlying the diminished TAp73 expression in MAPKi-resistant melanoma cells in comparison to the corresponding treatment-naive cells. According to our findings, one explanation can be offered by the assumption that the evolutionary pressure of the MAPK inhibition selects cells with reduced TAp73 levels correlating with a diminished genomic stability as a hallmark of cancer progression. This characteristic might offer an enhanced genomic flexibility and the generation of further genetic alterations that can confer the MAPKi-resistance mechanisms^44–46^. This assumption is in line with the reports of augmented *TP53* mutations during tumour progression mediating an enhanced genomic instability^47^. Nevertheless, we found that TAp73 knockdown does not influence the sensitivity towards short-time BRAFi treatment in in vitro assays. Similarly, the overexpression of TAp73 in MAPKi-resistant cells does not sensitize the cells towards BRAFi treatment. These results point out that the reduction of TAp73 levels upon MAPKi-resistance development specifically sensitizes melanoma cells towards platinum-based drugs but does not have a direct influence on the MAPKi sensitivity. Further analysis is required to clarify whether TAp73 reduction in MAPKi-resistant melanoma cells confers other growth advantages to these cells.

In summary, our data show that the expression level of TAp73 is critical for the sensitivity towards DNA crosslinking agents in melanoma cells, which is most likely independent of p53 activity. According to our data, we propose that the expression of TAp73 can be used as a marker predicting the susceptibility of MAPKi-resistant melanomas to platinum-based anti-neoplastic drug treatments.

## Electronic supplementary material

Suppl. Figure 1

Suppl. Figure 2

Suppl. Figure 3

Suppl. Figure 4

Suppl. Table

Suppl. Legend
