# Supplementary material for: Melanoma cells resistant towards MAPK inhibitors exhibit reduced TAp73 expression mediating enhanced sensitivity to platinum-based drugs
Source: Cell Death Dis. 2018 Sep 11;9(9):930. doi: 10.1038/s41419-018-0952-8 (PMC6133963; doi:10.1038/s41419-018-0952-8)
Supplement: Supplementary file 6 — Suppl. Legend [file 41419_2018_952_MOESM6_ESM.docx]

**Supplementary Figure 1**

**A.** Viability analysis after BRAFi (vemurafenib, up to 20 µM) or MEKi (trametinib, up to 250 nM) treatment for 72 h in S, R and DR melanoma cells. **B.** Viability assay after the treatment of S, R and DR melanoma cells with the indicated concentrations of 5-fluorouracil, temozolomide (TMZ) or paclitaxel. **A./B.** The viability signals were normalized to the non-treated cell signals. (Mean values ±SD; n=3).

**Supplementary Figure 2**

**A.** Treatments of ten metastatic melanoma patients (Pt1-Pt10) prior to the application of carboplatin/paclitaxel therapy (CP) are listed. **B.** Partial response of three patients (shown in A, Patient 1-3) after CP is reflected by the reduction of the tumour markers S-100 or LDH over time.

**Supplementary Figure 3**

**A.** Immunoblot analysis of p53, p21 and Mdm2 protein was performed in S and R melanoma cell lines. Actin served as a loading control. **B.** The picture on the left shows schematically the recognition sites of the p73 primer pairs within the first exons of the *TP73* gene for the detection of N-terminal p73 isoform expression (TAp73, ΔNEx2 p73, ΔNEx2/3 p73, ΔN´p73, ΔNp73). These primers were used for the experiments shown in Figure 2C and Supplementary Figure 3C and D. The picture on the right shows schematically the recognition sites of the p73 primer pair within the last exons of the *TP73* gene for the detection of C-terminal p73 isoform expression (α, β, γ, δ, ε, ζ). These primers were used for the experiments shown in Figure 2D. The numbered boxes indicate the different exons. Black arrows indicate the respective primer recognition sites. **C.** mRNA expression of different N-terminal p73 transcripts in R and S melanoma cells normalized to the corresponding *Actin* gene expression. The mRNA expression in corresponding S cells was set as 1. (Mean values ±SD; n=3) **D.** Relative distribution of different N-terminal p73 isoforms (in %) in S melanoma cell lines is shown. The respective mRNA expression normalized to *Actin* gene expression (n=3) was used for the analysis. **E**. Immunoblot analysis was performed using the anti-p73 5B429 antibody (left graph) described to selectively detect the class of TAp73 isoforms (Ref.9) and the anti-p73 H79 antibody (right graph) which is able to bind to all N- and C-terminal p73 isoforms. The arrows indicate the positions of TAp73α (left graph) or p73α (right graph). Actin served as a loading control.

**Supplementary Figure 4**

**A.** TAp73 mRNA expression (A.) was monitored in S melanoma cell lines 24 h after the transfection with siRNAs against TAp73 (si1 TAp73 or si2 TAp73) or control siRNA (siCtr.). *TA TP73* gene expression was normalized to the corresponding *Actin* expression and gene expression of untreated control siRNA transfected cells was set as 1. One exemplary experiment performed in triplicates is shown. **B.** Viability analysis was performed in S melanoma cell lines after the transfection of siRNA against TAp73 (si1 TAp73) or non-silencing control siRNA (siCtr.) (upper graph) as well as in R melanoma cells transfected with HA-TAp73α plasmid (TAp73α) or pcDNA3 control-plasmid (Ctr.) (lower graph) with or without additional vemurafenib treatment (up to 20 µM) for 72 h. Assay signals were normalized to the untreated cell signals. Exemplary data of one experiment with five replicates are shown (Mean values ±SD). **C.** Viability analysis after siRNA transfection (si1 TAp73, siCtr) with or without additional carboplatin (CBCDA; up to 250 µM) treatment for 72 h in S melanoma cell lines. Assay signals were normalized to the appropriate untreated cell signals. (Mean values ±SD; n=5) **D.** Shown is the percentage of apoptotic cells after siRNA transfection (si1 TAp73, si2 TAp73, siCtr) with or without additional carboplatin (CBCDA; 25 µM) treatment for 72 h. Data of one exemplary experiment performed in quintuplicates is shown **E.** Immunoblot analysis for p73, HA, p53 using R melanoma cell lysates collected 48 h after the transfection of HA-TAp73α (TAp73α) or control-pcDNA3 plasmid (Ctr). Actin served as a loading control. **F.** mRNA expression of genes involved in homologous recombination (HR) or nucleotide excision repair (NER). The samples were collected 24 h after transfection of the indicated S melanoma cells with siRNAs against TAp73 (si1 TAp73) or with control siRNA. Target gene expression was normalized to *Actin* expression and additionally to the corresponding gene expression of control siRNA transfected cells. (Mean values ±SD; n=3).

**Supplementary Table 1**

The sequences of the primers used in the RT-qPCR analysis are listed.
